# ZIP4 silencing improves bone loss in pancreatic cancer

**DOI:** 10.18632/oncotarget.4667

**Published:** 2015-07-20

**Authors:** Qiang Zhang, Xiaotian Sun, Jingxuan Yang, Hao Ding, Drake LeBrun, Kai Ding, Courtney W. Houchen, Russell G. Postier, Catherine G. Ambrose, Zhaoshen Li, Xiaohong Bi, Min Li

**Affiliations:** ^1^ Department of Orthopedics, General Hospital of the Jinan Military Command, Jinan, Shandong 250031, China; ^2^ The Vivian L. Smith Department of Neurosurgery, the University of Texas Medical School at Houston, Houston, TX 77030, USA; ^3^ Department of Gastroenterology, Changhai Hospital, Second Military Medical University, Shanghai 200433, China; ^4^ Department of Medicine, the University of Oklahoma Health Sciences Center, Oklahoma City, OK 73104, USA; ^5^ Department of Surgery, the University of Oklahoma Health Sciences Center, Oklahoma City, OK 73104, USA; ^6^ Department of Nanomedicine and Biomedical Engineering, the University of Texas Medical School at Houston, Houston, TX 77030, USA; ^7^ Department of Biostatistics and Epidemiology, College of Public Health, the University of Oklahoma Health Sciences Center, Oklahoma City, OK 73104, USA; ^8^ Department of Orthopedic Surgery, the University of Texas Medical School at Houston, Houston, TX 77030, USA

**Keywords:** ZIP4, bone, cachexia, pancreatic cancer, RANK/RANKL signaling

## Abstract

Metabolic bone disorders are associated with several types of human cancers. Pancreatic cancer patients usually suffer from severe nutrition deficiency, muscle wasting, and loss of bone mass. We have previously found that silencing of a zinc transporter ZIP4 prolongs the survival and reduces the severity of the cachexia *in vivo*. However, the role of ZIP4 in the pancreatic cancer related bone loss remains unknown. In this study we investigated the effect of ZIP4 knockdown on the bone structure, composition and mechanical properties of femurs in an orthotopic xenograft mouse model. Our data showed that silencing of ZIP4 resulted in increased bone tissue mineral density, decreased bone crystallinity and restoration of bone strength through the RANK/RANKL pathway. The results further support the impact of ZIP4 on the progression of pancreatic cancer, and suggest its potential significance as a therapeutic target for treating patients with such devastating disease and cancer related disorders.

## INTRODUCTION

Pancreatic cancer (PC) is one of the leading causes of cancer death worldwide with an incidence rate that is nearly comparable to its mortality rate [1]. The 5-year survival rate has remained less than 6% for the past three decades [1, 2], which demonstrates the aggressiveness and lethal nature of this disease. Clinical manifestations are usually non-specific, and patients commonly complain of abdominal pain, poor appetite, jaundice and wasting syndrome.

Wasting syndrome is also called cachexia, referring to the condition of deficient nutrition due to chronic or malignant diseases. A majority of PC patients develop severe cachexia with significant and progressive loss of adipose tissue and skeletal muscle mass [3–5]. The deterioration of musculoskeletal system in cachexia thus could result in increased morbidity such as immobility and mortality risk [6]. While the weight loss and muscle wasting are progressively visible during the course of tumor growth, little is known about the involvement of skeletal bone in cachexia despite its critical impacts on the long-term life quality of cancer patients. Bone mineral content was shown to be reduced significantly in lung cancer patients with 30% weight loss [7], which indicates that cachexia is associated with bone loss. Similar results were also reported in a preclinical animal model of lung cancer where concomitant loss of bone and muscle was observed [8]. In PC, the current research focus has been largely devoted to the clarification of the carcinogenesis and the development of potential therapeutics to inhibit the tumor growth, while rare studies have been conducted on the progression of cachexia in such patients, especially the bone changes. What's important; in clinical practice PC patients at advanced stage frequently develop cachexia-induced bone loss, leading to a high incidence of bone fracture. While muscle wasting has been currently under extensive investigation, bone loss and its related molecular mechanism in PC is still poorly understood. Furthermore, effective prevention and treatment of bone loss in these patients can improve their life quality, reduce both direct and indirect medical costs, and enhance the therapeutic efficacy against the primary disease.

Our previous studies have identified the cell membrane zinc transporter ZIP4 as a novel prognostic marker and therapeutic target for PC [9–11]. Zinc is an essential micronutrient and co-factor for many metalloenzymes and transcriptional factors [12, 13] and plays a critical role in tumor growth and metastasis [14, 15]. Dysregulated zinc transporters have been implicated in several human cancers [16–18]. We found that ZIP4 is overexpressed in majority of human PC, which correlates with tumor progression and survival *in vivo* [9, 10]. ZIP4 silencing could significantly suppress PC growth and reduce the cachexia syndrome in animal models [10], supporting that ZIP4 knockdown may offer a therapeutic benefit by improving the patients' overall health condition. In addition, RANK/RANKL (receptor activator of NF-κB ligand) signaling is the core in the regulation of the bone resorption, whether ZIP4 could mediate the bone metabolism in pancreatic ductal adenocarcinoma via RANK/RANKL pathway has never been examined ever before. Thus in this study, we aim to investigate the effect of ZIP4 silencing on the bone loss using orthotopic xenograft mouse model and further explore the related molecular mechanism.

## RESULTS

### Knockdown of ZIP4 improved the femoral microstructure and bone tissue mineral density

**Figure 1:**
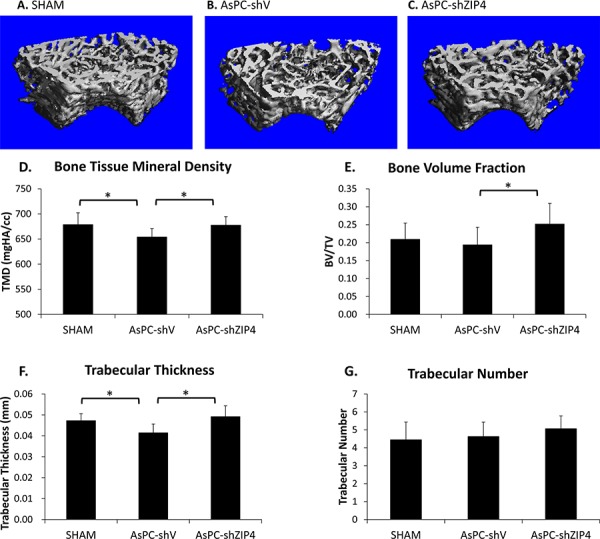
ZIP4 knockdown relieved bone resorption Representative 3-D Micro-CT images of trabecular bone microarchitecture above the growth plate of the distal end of the femur in SHAM **A**. AsPC-shV **B.** and AsPC-shZIP4 groups **C.** A volume of interest with 1.2 mm height was selected for the analysis of trabecular bone micro-architecture. All of the trabecular TMD of mice bearing pancreatic xenograft tumors of AsPC-1 cells presented an overall decrease. The femurs in the AsPC-shZIP4 group exhibited significant increase in the TMD and bone volume fraction as compared with that in the AsPC-shV group **D, E.** Qualitative analysis of trabecular bone revealed that silencing of ZIP4 in AsPC-1 cells (AsPC-shZIP4 group) could restore the decreased trabecular thickness, but exhibited no significant effect on the trabecular number **F, G.** *P < 0.05.

The results of typical micro-computed tomography (Micro-CT) analysis of femoral trabecular bones were shown in Figure 1A–1C. Pancreatic tumor bearing mice showed an overall decrease in trabecular bone tissue mineral density (TMD), with the mean TMD values being 678.7 ± 23.7 mgHA/cc, 654.0 ± 17.0 mgHA/cc, and 677.7 ± 17.0 mgHA/cc, for the SHAM, AsPC-shV, and AsPC-shZIP4 group, respectively (Figure 1D). The mice in AsPC-shV group retained 96.4% TMD, which is a statistically significant decrease (*P* = 0.04) compared to that of the SHAM group. The AsPC-shZIP4 mice showed a significant increase (*P* = 0.01) in TMD values compared to AsPC-shV mice, indicating a recovery of the bone mass with treatment. Bone volume fraction was lower in the AsPC-shV mice; this difference was not significant when compared to the SHAM animals, but was significant when compared to the AsPC-shZIP4 group (Figure 1E). Although the trabecular number was not significantly different among the groups, the bone architecture was affected as measured by trabecular thickness (Figure 1F–1G). The AsPC-shV mice demonstrated a significant decrease in trabecular thickness, which was restored by silencing ZIP4. Therefore, the silencing of ZIP4 in AsPC-1 cells (AsPC-shZIP4) exhibited significant reduction of trabecular bone loss and improvement of trabecular thickness. The cortical TMD in the femoral midshaft did not demonstrate significant differences among the three groups (data not shown).

### ZIP4 level was associated with bone composition changes

**Figure 2:**
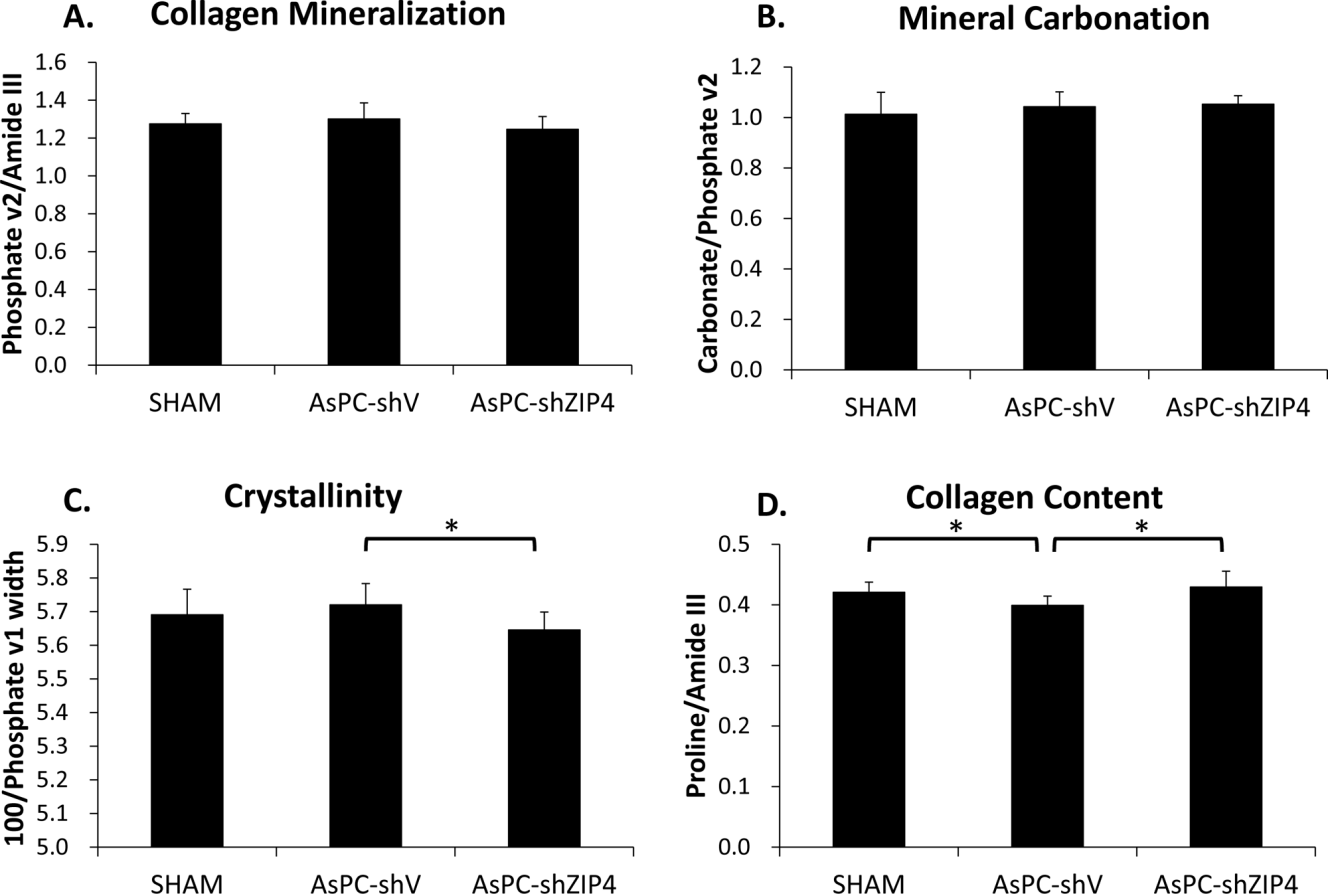
Bone composition analysis at distal metaphysis indicated that ZIP4 knockdown could optimize the bone composition No significant difference was found on the mineralization **A.** and carbonation **B.** among three groups (both *P* > 0.05). The crystallinity of the bones **C.** was significantly decreased and collagen content **D.** was increased in AsPC-shZIP4 group, respectively. *P < 0.05

To evaluate the response of bone constituents to tumor and treatments, Raman spectroscopy was utilized to further analyze bone composition in all groups. No significant differences were observed in collagen mineralization (mineral-to-matrix ratio) (Figure 2A) or mineral carbonation (Figure 2B) levels among the three groups. Mineral crystallinity as an indicator of crystal size and stoichiometric perfection [19–21], was significantly decreased with the treatment of ZIP4 knockdown (Figure 2C), predicting the presence of smaller or less crystalline mineral crystallites. Collagen content (Figure 2D), on the other hand, showed a significant increase in AsPC-shZIP4 mice, restoring the value to the SHAM group level. These results indicate elevated new bone formation in the treatment group.

### Effect of tumor burden on bone mechanics was abrogated by silencing ZIP4

**Figure 3:**
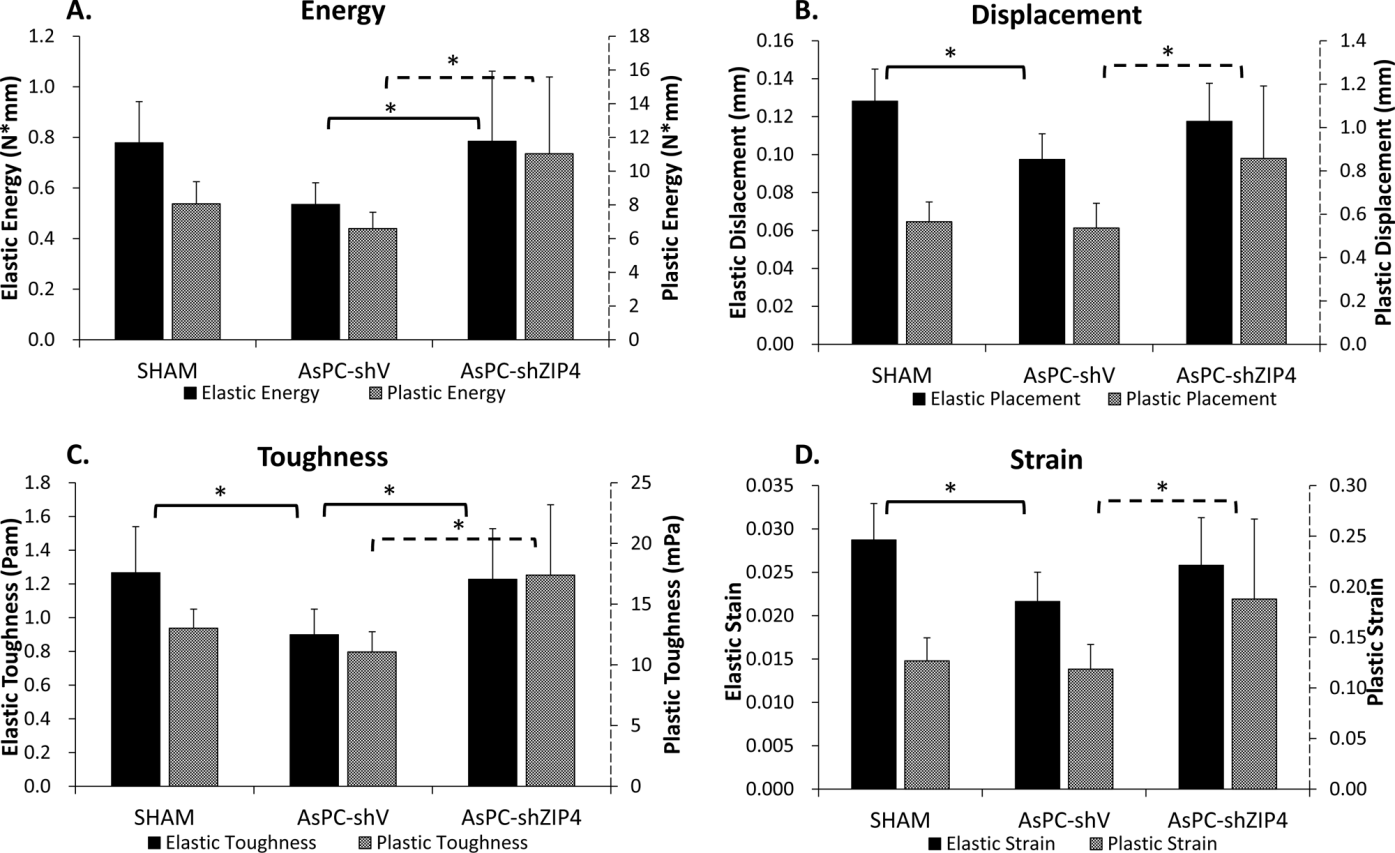
Mechanistic test results showed that ZIP4 silencing could improve mechanistic function of the bone The elastic and plastic energy **A.** plastic displacement **B.** elastic and plastic toughness **C.** and plastic strain **D.** were significantly increased in AsPC-shZIP4 group, when compared with AsPC-shV group (all *P* < 0.05). *P < 0.05

There were no significant differences among the groups for stiffness, yield load, ultimate load, elastic modulus, or ultimate strength (data not shown). Significant differences were observed among groups in elastic and plastic energy (Figure 3A), plastic displacement (Figure 3B), elastic and plastic toughness (Figure 3C), and plastic strain (Figure 3D) (all *P* < 0.05). For all the above mechanical test parameters with a significant difference, the lowest values were always present in the AsPC-shV group; and the values for the AsPC-shZIP4 treatment group were significantly higher than the AsPC-shV group yet not significantly different from the SHAM group (Figure 3A–3D). The improved mechanical properties in the ZIP4 silencing group suggest that the bones from these animals demonstrated higher ductility and could absorb more energy before failure.

### ZIP4 mediated the bone loss via the RANK/RANKL pathway

**Figure 4:**
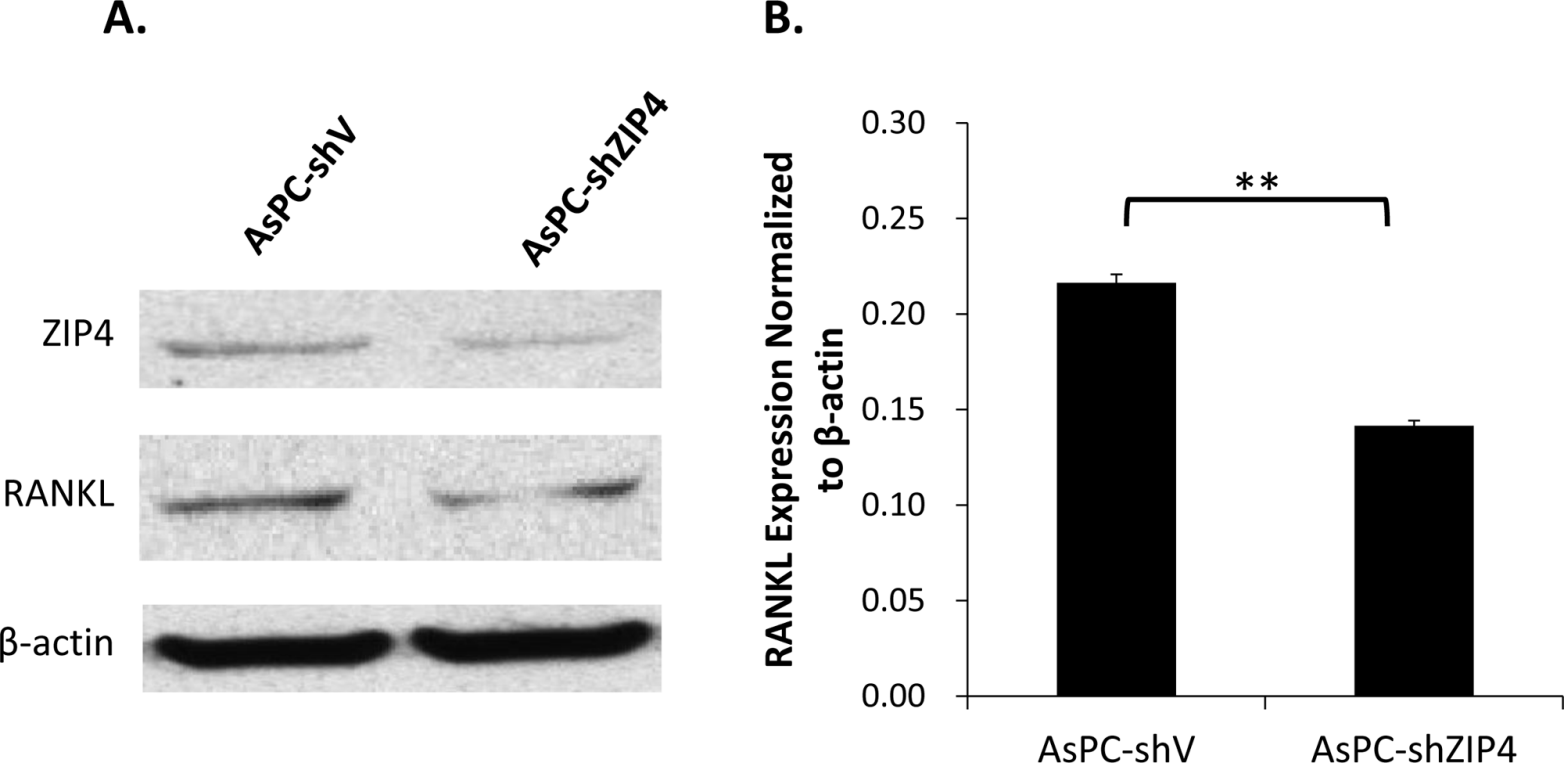
ZIP4 silencing down-regulated RANKL level under zinc-deficient condition Western blot showed that the knockdown of ZIP4 could attenuate the RANKL protein level in PC cells in Chelex zinc-chelating medium **A.** Quantification analysis confirmed that the expression level of RANKL normalized to β-actin was significantly decreased in AsPC-shZIP4 cells **B.** **P < 0.001

**Figure 5:**
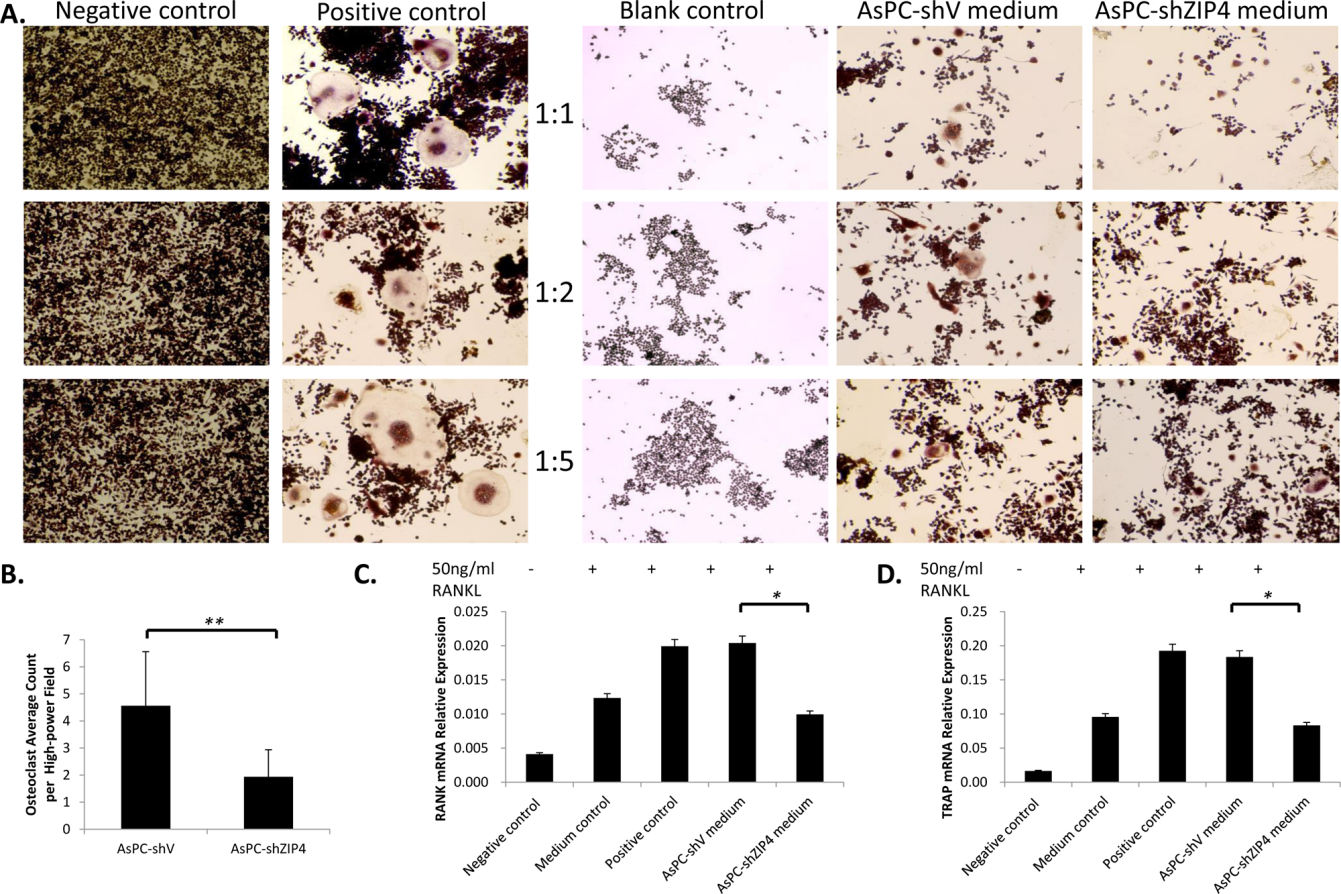
The differentiation of RAW264.7 cells to osteoclast was enhanced when culturing in the conditioned medium from AsPC-shV cells, compared with that from AsPC-shZIP4 cells Three dilution ratios (1:1, 1:2 and 1:5) of conditioned medium and complete medium with RANKL were examined, and further analysis were conducted under 1:2 conditioned medium. Osteoclasts were typically characterized by multinucleate, ruffled membrane and pseudopodia **A.** Culture in conditioned medium from AsPC-shV promoted the osteoclast differentiation (4.6 ± 1.9 vs. 1.9 ± 1.0, *P* < 0.001) **B.** 7,000 cells/well were reseeded into 24-well plate. Each treatment had four replication wells and the experiments were repeated three times. Four pictures were taken for each well and the average osteoclasts per high-power field (20X) were calculated. Treatment with conditioned medium could also regulate the level of RANK and TRAP mRNAs **C, D.** Exposure to AsPC-shV conditioned medium (1:2) upregulated the RANK and TRAP expressions. *P < 0.05, **P < 0.001

Several studies have already proved that RANK/RANKL signaling is the key in the regulation of osteoclast function and bone absorption [22–24]. To gain insight into the molecular mechanism of the ZIP4-induced bone loss, we explored the RANKL level in AsPC-shV and AsPC-shZIP4 cells and evaluated the influence of the conditioned medium from those two cell lines on the differentiation of murine macrophage RAW264.7 cells into osteoclasts. Western blot analysis showed that RANKL level in AsPC-shV cells was higher than that in AsPC-shZIP4 cells under zinc-deficient environment (Figure 4A–4B). Culture in the medium from AsPC-shV cells increased the average differentiated osteoclast count per high-power field (4.6 ± 1.9 vs. 1.9 ± 1.0 per high-power field, *P* < 0.001) (Figure 5A–5B), and upregulated the RANK and TRAP mRNA levels in differentiated RAW264.7 cells induced by RANKL treatment (Figure 5C–5D). These data suggest that RANK/RANKL pathway may be involved in the ZIP4-mediated bone loss seen in PC patients.

## DISCUSSION

Zinc transport and homeostasis are critical in tumor growth and progression in PC [9–11]. Recent studies in our group revealed that silencing of zinc transporter ZIP4 not only inhibits PC growth, but also decreases the symptoms of cancer cachexia such as muscle wasting and weight loss. However, little has been known on the effect of ZIP4 on the bone in PC, although bone disorders in PC patients are common in clinical practice. Here our study further investigated the cachexia-associated alterations in the skeletal bone of PC and evaluated the effect of ZIP4 silencing on bone deterioration.

Bone loss in the tumor bearing mice has been observed mainly in the trabeculae, where TMD was reduced by about 4% compared to the controls. The extent of bone loss is in agreement with the whole body TMD reduction (around 5–6%) in a preclinical animal model of lung cancer [8]. Our results showed that no significant differences are observed in cortical TMD or collagen mineralization in femoral cortex. The overall material properties of bone at the tissue level are impaired as demonstrated by the deteriorated mechanical function, indicating the importance of the under-explored bone deterioration to PC management. To the best of our knowledge, the present work may be the first report of bone quality alteration in ZIP4 related PC.

Our studies of ZIP4 silencing have shown the restoration of trabecular TMD and mechanical properties besides the previously reported weight improvement [10], suggesting the involvement of ZIP4 in the regulation of muscle and bone mass in cachexia. In our previous study, the activation of NF-κB has been found significantly decreased with the silencing of ZIP4 [10], indicating that NF-κB signaling might have been regulated through ZIP4 down-regulation. NF-κB is critical in the pathology of muscle wasting [25, 26]. NF-κB activation in muscle through IκB kinase (IKK) induces severe muscle wasting syndrome in mice, while the blockade of NF-κB reverses the phenotype of muscle atrophy [27]. Treating MAC16 tumor bearing mice with resveratrol, which inhibits the pathway of IKK/NF-κB, significantly reduced muscle loss, confirming the importance of NF-κB inhibition in cachexia treatment [28]. Meanwhile, abundant evidence suggests the crucial role of NF-κB signaling in skeletal development and the functions of osteoclast and osteoblast [29–32]. The specific inhibition of IKK/NF-κB suppressed inflammatory bone loss in an arthritis model by inhibiting osteoclastogenesis [29], supporting NF-κB as a potential target for treating pathological bone resorption. These reports are consistent with the effect of ZIP4 silencing on bone disorder in the current study. Our findings reveal that ZIP4 may exert concomitant regulation on muscle and bone mass through the NF-κB signaling.

**Figure 6:**
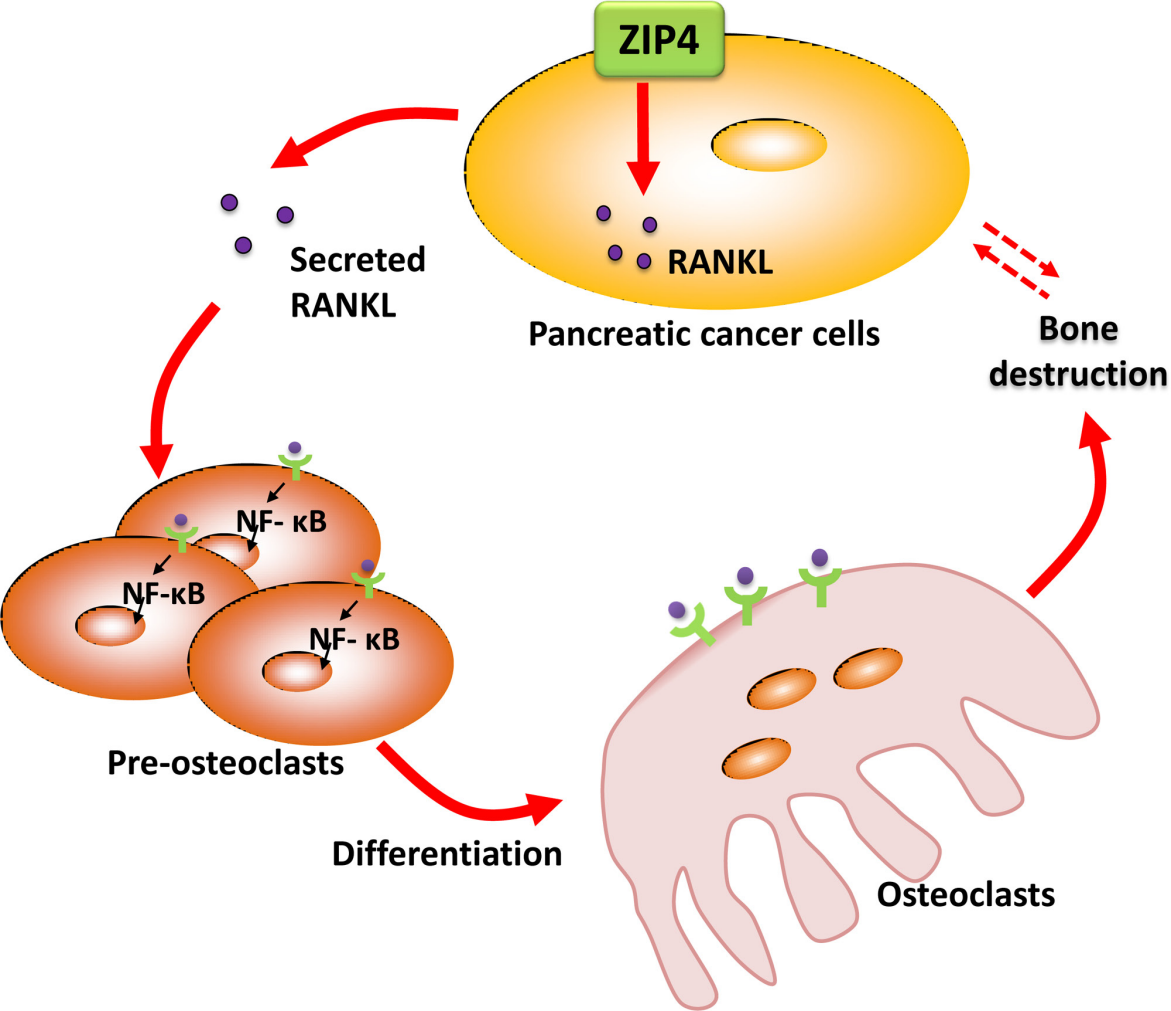
Diagram for ZIP4-induced bone loss in pancreatic cancer ZIP4 knockdown in pancreatic cancer could regulate the bone metabolism and structure via the RANK/RANKL pathway.

Our studies of RANK/RANKL provide further evidence for the association between ZIP4 level in PC tumor and the NF-κB signaling in bone. RANK/RANKL signaling is a typically critical regulator in the activity of osteoclast [33], which is shown to be associated with bone disorders, including those related to human malignant diseases. RANKL is a member of tumor necrosis factor (TNF) superfamily, which has two forms: transmembrane protein and secreted protein [34]. The binding of RANKL to RANK on the membrane of osteoclasts promotes bone resorption via activating the osteoclasts. Activation of NF-κB is a crucial target of RANK/RANKL signaling [35, 36], inducing osteoclast formation. In breast cancer, RANK/RANKL signaling is required for cancerous mammary epithelial cell proliferation, and RANK-deficient mice manifest a markedly delayed hormone- and oncogen-driven breast carcinogenesis [37]. In prostate cancer, RANKL expression is positively associated with tumor growth and metastasis [38]. However, its role in PC has not been well studied. In the current study, the expression of RANKL in tumor cells is modulated with the level of ZIP4. Thus, we propose a RANKL-induced osteoclast differentiation model here to elucidate the PC tumor-bone interaction. As illustrated in Figure 6, ZIP4 could regulate the expression of RANKL, leading to the dysregulation of RANK/RANKL pathway through IKK/NF-κB signaling in osteoclasts in the bone tissue of PC tumor bearing animals. Considering that the control system of bone metabolism is rather complex involving multiple signal transduction pathways, cross-link between RANK/RANKL signal and other aberrant pathways need to be further verified.

In summary, we first proposed that silencing ZIP4 could alleviate the bone destruction in PC xenograft mouse model, and went deep into the molecular mechanism that ZIP4 may regulate the RANK/RANKL signaling via releasing RANKL into circulation which transmits from primary tumor sites to bone lesions. All these results support that ZIP4 exerts an extensive effect on pancreatic carcinogenesis and could be a therapeutic target for treating the primary tumor as well as cachexia in skeletal muscle and bone in PC patients.

## MATERIALS AND METHODS

### Cell culture and chemicals

Human PC cell lines AsPC-1 were purchased from the American Type Culture Collection (ATCC, Rockville, Maryland, USA), and were authenticated by DNA finger printing in April, 2009. The establishment of control scramble and ZIP4 specific shRNA expressing stable cell lines (AsPC-shV, AsPC-shZIP4) were previously described and cultured in complete RPMI 1640 medium containing 1% penicillin-streptomycin, 1% sodium pyruvate and 10% FBS [39]. Murine macrophage RAW264.7 cells were purchased from ATCC, and cultured in complete DMEM medium with 1% penicillin-streptomycin, 1% sodium pyruvate and 10% FBS.

### Orthotopic xenograft mouse model

Subconfluent cells (3 × 10^6^) were inoculated into the tail of the pancreas of 5- to 6-week-old male nude mice (Hsd: Athymic Nude-Foxn1nu, Harlan Lab). Mice in SHAM group underwent the surgical procedures but no tumor cells were injected. All mice were cared for in accordance with the Office for Protection from Research Risks (OPRR) and Animal Welfare Act Guidelines under an animal protocol approved by the institutional Animal Welfare Committee. The animals were euthanized at 25 days post tumor inoculation, and both femurs were dissected for further analysis.

### Micro-CT

Right femur samples were put into a 16 mm tube filled with PBS and scanned at 16 micron resolution using a Scanco μCT40 scanner (SHAM group: *n* = 6, AsPC-shV group: *n* = 8, AsPC-shZIP4 group: *n* = 8). Image reconstruction and analysis were performed using the Scanco software by an experienced blinded orthopedic surgeon. The femoral diaphysis and distal trabeculae were manually selected (contoured) every five slices, and then the remaining slices were morphed to enclose the region of interest, for a total of 75 slices (1.2 mm) with threshold at 210 and a gauss setting of 0. Bone TMD and trabecular bone micro-architecture were quantified based on the mean TMD of midshaft cortical bone, and the trabecular parameters such as bone volume fraction (bone volume over total volume, BV/TV), trabecular number and thickness.

### Raman spectroscopy

Raman spectroscopic measurement was performed on left femurs using a confocal Raman microspectroscope (Invia Raman Microscope, Renishaw Inc., Gloucestershire, England) as described previously [40–42]. In brief, Raman spectra were collected from the distal end of femoral cortex which was leveled horizontally on Raman microscope stage. Thirty milliwatts of 785 nm laser was focused on the cortical surface through a Leica 50X/0.50 objective. The scattered Raman signals were collected through the same objective, coupled to a spectrometer, and detected by a CCD camera. Three Raman measurements were conducted and averaged for each femur. Spectral parameters related to bone signatures such as the abundance (peak height) of phosphate (ν1, 960 cm^−1^; ν2, 436 cm^−1^), carbonate (1070 cm^−1^), proline (856 cm^−1^) and protein amide III (1256 cm^−1^) were calculated using custom written scripts in MATLAB. The amount of collagen mineralization and mineral carbonation were determined by the peak height ratios of phosphate ν2/Amide III and carbonate/phosphate ν2, respectively, to minimize the effect of orientation and system polarization [43–47]. Collagen content was quantified by taking the peak height ratio of proline and amide III. The crystallinity of mineral was assessed by the reciprocal of the full width of phosphate ν1 band at half of the maximum intensity (FWHM).

### Biomechanical testing

Femurs were tested in three point bending using a span of about 6 mm (5.96 mm) using an Instron 5848 device (Instron Inc., Norwood, Massachusetts, USA). All the femurs were tested wet at room temperature. They were preloaded to 1 N at a rate of 0.2 N/s for 5 seconds and then were compressed to failure at a rate of 0.1mm/s. Load and displacement data were captured at rate of 40 Hz by using Bluehill software (Instron 5848). A MATLAB program was created to analyze the load-displacement data files to extract the yield point, maximum load, and stiffness using a 0.2% offset strain. The elastic region was identified as the region from the completion of the preload to the yield point and the plastic region was identified as the region from the yield point until the point at which the change in load exceeded −40 N/s, indicating failure. Elastic and plastic energies were calculated as the areas under the elastic and plastic regions of the load-displacement curve. BoneJ was used to analyze the femoral midshaft Micro-CT images to obtain geometric data (cross-sectional moment of inertia and AP diameter) [48]. Stress and strain were calculated from the load-displacement data using the following formulas:

$$\text{Stress}=\frac{(0.5h)\text{FL}}{4I}\;\text{Strain}=\frac{6\;\text{Dh}}{L^{2}}$$

where F is the load, L is the span length, h is the specimen diameter, I is the cross-sectional moment of inertia, and D is the actuator displacement in mm. The same methods described above were used to determine the following parameters from the stress-strain curve: ultimate strength, elastic modulus, and toughness.

### Western blot

AsPC-shV and AsPC-shZIP4 cells (6 × 10^6^) were seeded into 100 mm dishes, respectively. After being treated with 0.5% FBS 2% Chelex 100 (Bio-Rad) medium which chelates the zinc, and 0.5% FBS medium for 24 h, cells were collected and lysed. Bicinchoninic acid (BCA) assay kit (Thermo Fisher Scientific) was used to evaluate the protein concentrations of the cell total lysates. 30 μg denatured protein samples were loaded in precast Bolt^®^ Bis-Tris Plus Gel (Life Technologies). The blot was first blocked in 5% milk TBST, and then incubated in 1:1000 anti-RANKL antibody (Santa Cruz), 1:1000 homemade ZIP4 polymonal antibody [11] and 1:4000 β-actin (Santa Cruz) 5% milk TBST overnight. Enhanced Chemiluminescent (ECL) or SuperSignal West Pico Chemiluminescent Substrate (Thermo Fisher Scientific) was used to detect the blot. The protein expression was normalized to β-actin and quantified by Image Studio Lite version 4.0 software.

### Osteoclast differentiation assay

RAW264.7 cells (7,000/well) were seeded in 24-well plate and the medium with or without 50 ng/ml RANKL (PeproTech) was changed every two days and this working concentration of RANKL was optimized by gradient pretests (Supplementary Figure 1). Negative control (complete DMEM medium without RANKL), positive control (complete DMEM medium with 50 ng/ml RANKL); blank control (1:1, 1:2 and 1:5 mixture of complete DMEM and RPMI 1640 medium with 50 ng/ml RANKL); AsPC-shV conditioned medium (1:1, 1:2 and 1:5 mixture of complete DMEM and AsPC-shV medium with 50 ng/ml RANKL); and AsPC-shZIP4 conditioned medium (1:1, 1:2 and 1:5 mixture of complete DMEM and AsPC-shZIP4 medium with 50 ng/ml RANKL) were maintained for 7 days. Each treatment had four different replication wells and the experiments were repeated for three times. Cells were fixed and stained by TRAP (Tartrate-resistant acid phosphatase) staining kit (Sigma). Four pictures were taken for each well and the average TRAP-positive cells per high-power field (20X) were counted and calculated.

### Detection of RANK and TRAP mRNA levels

Total RNAs of RAW264.7 cells treated under 1:2 conditioned medium as mentioned above were extracted using Purelink^®^ RNA mini kit (Life technologies). Turbo™ DNase (Life technologies) was used to remove the genomic DNA and cDNA was synthesized using High-Capacity cDNA Reverse Transcription kit (Life technologies) with 2 μg total RNA. Real-time PCR were performed to detect the relative expression level of RANK and TRAP, using β-actin as internal control.

### Statistical analysis

All statistical analyses were performed using SPSS 17.0 software (SPSS, Chicago, Illinois, USA). Continuous data are presented in the paper as mean ± standard deviation (SD). To compare each variable in Micro-CT, composition, mechanical analysis, and RANKL and TRAP mRNA levels among different groups including control (SHAM), AsPC-shV and AsPC-shZIP4 groups, one-way analysis of variance (ANOVA) with Turkey post-hoc comparison was used. The statistical analysis was performed by the Student's *t* test between AsPC-shV and AsPC-shZIP4 groups. *P* value < 0.05 was considered to be statistically significant.
